# Dystocia in the Standardbred Mare: A Retrospective Study from 2004 to 2020

**DOI:** 10.3390/ani12121486

**Published:** 2022-06-08

**Authors:** Aliai Lanci, Francesca Perina, André Donadoni, Carolina Castagnetti, Jole Mariella

**Affiliations:** 1 Department of Veterinary Medical Sciences, University of Bologna, Via Tolara di Sora 50, Ozzano dell’Emilia, 40064 Bologna, Italy; carolina.castagnetti@unibo.it (C.C.); jole.mariella2@unibo.it (J.M.); 2 Independent Researcher, Località Caiar 25, Caprino Veronese, 37013 Verona, Italy; francescaperina27@gmail.com; 3 Independent Researcher, Via Frittaia 30/A, Casaleone, 37052 Verona, Italy; donadoniandre@gmail.com; 4 Health Science and Technologies Interdepartmental Center for Industrial Research (CIRI-SDV), University of Bologna, 40100 Bologna, Italy

**Keywords:** dystocia, equine, Standardbred mare, foal health, foaling difficulty, categories of dystocia severity, causes of dystocia, foal blood parameters

## Abstract

**Simple Summary:**

In the equine species, dystocia, any impediment to normal parturition, is not frequent, but when it occurs it can quickly evolve into a critical situation. The aim of this study was to retrospectively describe the incidence, causes, categories of dystocia severity, resolution procedures and postpartum complications in Standardbred mares hospitalized in a Veterinary Teaching Hospital and evaluate the effects of dystocia on the clinical and blood parameters of their foals. In normal pregnancy, the incidence of dystocia was 4.9%. Stage II appeared significantly longer in dystocic delivery, and the occurrences of postpartum complications in mares and onset of neonatal disease in foals were more frequent after dystocia. This study also investigated, for the first time, foaling difficulty, dividing all dystocic deliveries into mild, moderate and severe dystocia. Therefore, even a short but physiologically abnormal delivery can pose a risk to the life of the newborn and determine complications in the mare.

**Abstract:**

Dystocia as a prolonged stage II parturition (>30 min) was associated with a higher risk of complications. The hypothesis of the study was that any type of dystocia could affect the foal’s health, even when the stage II was <30 min. Clinical reports on 222 Standardbred mares and their foals hospitalized at the Veterinary Teaching Hospital of the University of Bologna from 2004 to 2020 were reviewed. Mares were divided into the Eutocia Group (165, eutocic delivery) and the Dystocia Group (57, dystocic delivery). The incidence of dystocia was 4.9%. Stage II was longer in the Dystocia Group (median 20 min) than in the Eutocia Group (median 12 min). All occurrences of dystocia were retrospectively classified into three categories of severity: mild, moderate and severe dystocia. The occurrence of postpartum complications in mares and neonatal diseases and failure of passive transfer of immunity in foals was higher in the Dystocia Group. Foal venous lactatemia and serum creatine kinase were significantly higher in the Dystocia Group (median 3.9 mmol/L; 262 UI/L respectively) than in the Eutocia Group (median 3.1 mmol/L; 187 UI/L respectively). The APGAR score was lower in the Dystocia Group (median 8) than in the Eutocia Group (median 10) and significantly lower in severe dystocia (median 3). The duration of stage II should not be considered the only parameter of dystocia in mares: even a rapid resolution of dystocia could pose health risks to the foal and the mare.

## 1. Introduction

In the mare, dystocia has been described as any impediment to normal parturition that could result from maternal, fetal and/or fetal membrane causes [1,2,3]. It is a rare event, but when it occurs it can quickly evolve into a critical situation and lead, in the absence of intervention, to the death of the foal and potentially of the mare [4]. Prompt and correct obstetrical intervention could preserve not only the health of the foal but also the life and the fertility of the mare [2,3,4,5,6,7,8]. In Thoroughbred and Standardbred mares the reported incidence is 4%, in Belgian draft horses 10%, and in Shetland ponies 8% [5]. Ginther and Williams [6] reported prevalences of 16%, 10.5%, 8.9% and 19% for the Quarter horse, Standardbred, Thoroughbred and Miniature horse respectively, while McCue and Ferris [7] reported incidences of 10% in Thoroughbreds and Quarter horses. There is only one retrospective study carried out exclusively in the Standardbred, which reported an incidence of 2.7% [9]. The most common cause is faulty fetal disposition rather than maternal causes [6]. Approximately 98% of foals adopt an anterior longitudinal presentation by 8.5 months of gestation [10]. The foal normally assumes a dorsopubic position with hindlimbs encased in one uterine horn, preventing it from reverting to a posterior presentation [11]. During the last month of pregnancy, the fetus can change its position only along its longitudinal axis by rotation between the dorsoilial and dorsopubic positions [11,12]. During parturition, active fetal rotation in the dorsoilial position can be clockwise or counterclockwise, and the extension of the head and the forelimbs, which are the normal posture, represent the key events of a normal parturition and are completed during stage II [11,12]. It is widely recognized in the literature and in equine practice that the duration of stage II in eutocic delivery should be 20 to 30 min [12]. The length of stage II can be influenced by the type of dystocia, the experience of the veterinarian and the facilities (whether dystocia occurred on a farm or in a veterinary hospital).

Common complications after dystocia include reproductive tract trauma, retained fetal membranes, and delayed uterine involution. Further complications include uterine artery hemorrhage, uterine prolapse, neuropraxis, and pressure necrosis. These complications may become evident from immediately postpartum to several days after [13].

A retrospective study at a referral hospital indicated that every 10 min increase in the duration of stage II beyond 30 min is associated with a 10% increase in the existing risk of a foal being stillborn and a 16% increase in the risk of the foal not surviving until discharge from the hospital [8]. A prolonged stage II is associated with fetal asphyxia, hypoxia, and a higher risk of foal mortality [14]. To the authors’ knowledge, there are only two studies on foals of draft breeds that evaluate the consequences of dystocia on foals’ clinical parameters [14,15].

The aim of the present study was to retrospectively describe incidence, causes, degree of dystocia severity, type of resolution and postpartum complications in Standardbred mares hospitalized at a Veterinary Teaching Hospital, and to evaluate the effects of dystocia on the clinical and blood parameters of their foals. The hypothesis was that dystocia could also affect foal health when, with corrective obstetric procedures, stage II lasted less than 30 min.

## 2. Materials and Methods

### 2.1. Population and Data Collection

All medical reports regarding 222 Standardbred mares hospitalized at the Equine Perinatology and Reproduction Unit (Equine Clinical Service, Veterinary Teaching Hospital of the Department of Veterinary Medical Sciences, University of Bologna, Italy) from 2004 to 2020 were reviewed. Mares hospitalized for attended delivery and mares referred for dystocia after the onset of stage II were included. Data recorded at admission and during hospitalization of mares and foals were collected from clinical reports and the hospital veterinary medical information system (FeniceVET^®^, ZakSoft srl, Bologna, Italy).

Mares hospitalized for assisted parturition were admitted at approximately 310 days of gestation and remained under around-the-clock observation until at least 7 days postpartum. The mares were housed in separate wide straw-bedded boxes with night vision cameras, fed hay ad libitum and concentrates twice a day, and were allowed to go to pasture during the day. All mares received a complete physical examination twice a day during hospitalization and a complete blood cell count and blood chemistry at admission. Additionally, transrectal palpation and ultrasonographic examination were performed to evaluate the combined thickness of the uterus and placenta (CTUP), fetal presentation and vitality, and quality of fetal fluids at admission and every ten days until parturition. In case of suspected high-risk pregnancy, a transabdominal ultrasonographic examination was performed to evaluate CTUP and fetal biophysiologic profile. The reference ranges of CTUP were considered in relation to gestational age, as reported elsewhere [16,17]. Type of pregnancy (normal or high-risk pregnancy) was recorded. High-risk pregnancy was defined by the occurrence of a history of premature udder development/lactation, increase in combined thickness of the uterus and placenta, purulent/serosanguineous vulvar discharge and/or systemic illness in the mare [18,19]. On the basis of the diagnosis, appropriate treatment was performed.

All parturitions were assisted by one or two experienced veterinary obstetricians. If parturition was eutocic it was assisted without intervention. The time of the rupture of chorioallantois was recorded; after the appearance of the amniotic membrane through the vulva, amniotic fluid was collected via needle puncture using a 60-mL sterile syringe [20,21], and amniotic lactate concentration (mmol/L) (Lactate SCOUT+, SensLab, GmbH, Lepzig, Germany) [20] was measured.

Dystocia was defined as any impediment to normal delivery due to complications of maternal, fetal and/or fetal membrane origin [1,2,3]. If there was no rupture of the chorioallantois, the veterinarian examined the birth canal. If an intact chorioallantois in the birth canal was found, it was immediately cut with a scalpel to allow for subsequent normal parturition. If within 5 min of chorioallantois rupture the amniotic vesicle did not emerge, the veterinarian examined the birth canal. If by 15 min after the chorioallantois rupture and the appearance of the amniotic vesicle, the forelimbs and head of the foal did not emerge, examination of the birth canal and presenting fetus was performed. If a faulty foal disposition could be corrected manually or with the use of obstetrical instruments, an assisted vaginal delivery (AVD) was performed. If the AVD was unsuccessful within 15 min, a controlled vaginal delivery (CVD) was performed with the mare under general anesthesia in dorsal recumbency with suspended hindlimbs. Typically, the CVD would take place in the induction box of the Equine Surgery Unit since, if it was not possible to resolve the dystocia within 20–25 min, a cesarean section was performed.

The obstetrical intervention in mares referred for dystocia depended on the previous intervention performed at the breeding farm or in the field. If the reproductive tract of the mare was already traumatized, the mare went directly to CVD or cesarean section.

Finally, the dystocia resolution procedure was recorded: AVD, CVD, fetotomy or cesarean section [22].

Regarding foaling difficulty, all dystocias were retrospectively classified into three categories of severity:

✓ “mild” when a limited intervention with AVD was sufficient for resolution, such as in the case of slight traction performed by one or two people, the administration of oxytocin, the opening of the chorioallantois, or episiotomy; oxytocin was administered (10/20 UI IV) only in case of normal fetal disposition when the uterine contractions were not effective [13,23];
✓ “moderate” when obstetrical procedures were prolonged in terms of degree of traction that required the use of obstetrical instruments, as in the case of severe fetal maldisposition or malformation (AVD);
✓ “severe” when procedures other than AVD were used, such as CVD, cesarean section or fetotomy.

During dystocia, if the foal’s nose was accessible, the ex-utero intra-partum treatment (EXIT) procedure was performed using a cuffed endotracheal tube, a self-inflating bag-valve device and oxygen [24].

The following data were recorded for all mares: age, parity, type of pregnancy (normal or high-risk), duration of stages II and III (min), cause of dystocia (maternal, fetal and/or fetal membrane), categories of dystocia severity (mild/moderate/severe), dystocia resolution procedure, mare blood lactate (mmol/L) (Lactate SCOUT+, SensLab, GmbH, Lepzig, Germany), gross fetal membrane alterations, umbilical cord length, total umbilical coil number, umbilical coiling index (UCI: umbilical coils divided by umbilical cord length) [25,26]. Umbilical cord assessment (coils, length and UCI) was collected beginning in 2012. All mares and foals after foaling were closely monitored and received a complete physical examination every 6 h. Postpartum complications were recorded. Retained fetal membranes (RFM) were diagnosed 3 h after parturition [27,28].

The following data were collected from all foals at birth: weight (kg), sex, APGAR score [29], temperature, glycemia (glucometer, Medisense Optium; Abbott Laboratories, Medisense Products, Bedford, MA, USA), jugular blood lactate (Lactate SCOUT+, SensLab, GmbH, Lepzig, Germany), venous blood gas analysis parameters (VetStat Electrolyte and Blood Gas Analyzer, Idexx, Westbrook, ME, USA), cell blood count (CBC) (from 2004 to 2009 via CELL-DYN3500R, Abbott Laboratories, Santa Clara, CA, USA; from 2009 to 2020 via ADVIA 2120, Siemens Healthcare srl, Milan, Italy) and blood chemistry parameters (Chemistry Analyzer AU400; Olympus Diagnostica GmbH, Lismeehan, Ireland). Blood–biochemistry profiles changed over the years depending on the instruments used and the requests of the veterinary clinicians.

Colostrum was evaluated after birth with a brix refractometer and the first suckling was assisted in order to occur within 2 h after birth. Then, normal suckling was assisted every hour. In foals that were not able to suckle or not able to remain standing, colostrum was administered through a nasogastric tube. If the brix value of the maternal colostrum was less than 20% [30,31], high quality colostrum from a frozen colostrum bank (brix >25%) was thawed at <37 °C and administered to the foal with nasogastric tube. Each foal received almost 1 L of colostrum of good quality within its first 8 h of life.

Time of onset of suckling reflex (min from birth), time to acquire sternal recumbency (min from birth), standing position (min from birth) [32], first intake of colostrum (min from birth), IgG concentration at 12–24 h (from 2004 to2009 via SNAP Foal IDEXX, Milan, Italy; from 2009 to 2020 via DVM Rapid Test II immunoturbidimetric test, MAI Animal Health, Elmwood, WI), and clinical diagnosis [19,33] were recorded. Regarding the type of medical assistance to foals, the level of care needed, from 1 to 3 (Level 1: minimum assistance; Level 2: additional assisted feeding; Level 3: intensive care), was recorded [34]. For both mares and foals, outcomes were recorded, and mortality rate was considered up to seven days postpartum.

Foals were classified as healthy when they had an APGAR score at birth ≥9, a normal clinical evaluation during the course of hospitalization, including complete blood count and serum chemistry at birth, and an IgG serum concentration >800 mg/dL at 12–24 h of life.

Mares were divided into a Eutocia Group and a Dystocia Group based on the type of foaling. To evaluate the effect of dystocia on foals’ clinical and blood parameters, the foals of the Eutocia Group were further divided into a Control Group that included only healthy foals born from normal pregnancy after eutocic delivery. Foals of the Dystocia Group were then compared with the Control Group regarding clinical parameters and rapid determinations performed at foaling, venous blood gas analysis, CBC and blood chemistry parameters.

### 2.2. Statistical Analysis

Data distribution was analyzed using the Kolmogorov–Smirnov test. Since data were not normally distributed, non-parametric tests were used and data were expressed as medians and interquartile range (IQR). Spearman’s correlation test was used to evaluate the presence of correlations among clinical, blood gas and blood chemistry parameters. Differences among groups (Eutocia vs. Dystocia Group and Control Group vs. Dystocia Group) were analyzed with Mann–Whitney and Kruskal–Wallis tests for the quantitative variables. Categorical variables were tested with the chi-square test (χ^2^). A *p* < 0.05 was considered statistically significant. All statistical analyses were carried out using the commercial software Analyse-it, version 5.68 (Analyse-it Software Ltd., Leeds, West Yorkshire, UK).

## 3. Results

Due to the retrospective nature of the study, some data are missing, especially in earlier years when the hospital’s veterinary medical information system^a^ and paper-based medical reports were not completed.

### 3.1. Clinical Parameters in the Eutocia and Dystocia Groups

The study included 222 Standardbred mares; 217/222 (98.2%) mares were hospitalized for attended delivery at approximately 310 days of pregnancy, while 5/222 (1.8%) mares started foaling at the farm and were referred for dystocia. Data regarding mares are reported in Table 1.

Mares were divided into the Eutocia Group, including 165/222 (74.3%) mares with eutocic delivery, and the Dystocia Group, including 57/222 (25.7%) mares with dystocic delivery. Considering mares hospitalized for attended parturition, the incidence of dystocia was 24% (52/217 mares).

High-risk pregnancies totaled 32/222 (14.4%) and included placentitis (6), placental edema and placental abruption (16), abdominal ventral hernia (1), rupture of the prepubic tendon (1), and systemic illness in the mare (8). Among only the mares with high-risk pregnancies (32/222), the incidence of dystocia was 47% (15/32). Among only the mares with normal pregnancy (185/222), the incidence of dystocia was 4.9% (9/185). In the Eutocia Group, 58/165 (35%) were primiparous and 107/165 (65%) were multiparous.

Fifty-five (24.7%) mares presented postpartum complications. In the Eutocia Group, retention of fetal membranes (RFM) was the most frequent complication (10/165, 6%), followed by vaginal hematoma (8/165, 5%), perineal lacerations (6/165, 4%), puerperal septic metritis (4/165, 2%), constipation (2/165, 1%), laminitis (2/165, 1%) and cervical laceration (1/165, 0.6%). In the Dystocia Group, postpartum complications included RFM (13/25, 52%), vaginal hematoma (2/25.8%), puerperal septic metritis (2/25.8%), cecal rupture (2/25.8%), hemorrhage (1/25, 4%), cervical laceration (1/25, 4%), uterine prolapse (1/25, 4%), mastitis (1/25, 4%) and constipation (1/25, 4%). Mares that died of postpartum complications totaled 5/222 (4%), all belonging to the Dystocia Group. The causes of death were the rupture of the cecum in 2/5 mares, while 3/5 were subjected to humane euthanasia due to complications resulting from ventral abdominal wall hernia, uterine prolapse and postpartum hemorrhagic shock, respectively.

All foals from the Eutocia Group mares were born alive (165/165) while in the Dystocia Group there were 8/57 (14%) stillborn. The stillbirth rate of the total population was 3.6% (8/222). Data regarding sex and weight of the foals of both Groups are reported in Table 1. No difference was found in foal weight between males and females and between the two groups. The frequency of dystocia was not related to the sex of the foal. In the Eutocia Group, the diagnoses of sick foals were 19/20 (95%) neonatal encephalopathy and 1/20 foal (5%) had sepsis. Among the foals with neonatal encephalopathy, 7/19 (37%) were born from normal pregnancies but with gross fetal membrane alterations and 6/19 (32%) were born from high-risk pregnancies. The remaining 6/19 foals with neonatal encephalopathy were born from normal pregnancies and did not show gross fetal membrane alterations. In the Dystocia Group, there were 25/49 (51%) sick foals, of which 12/25 (48%) were born from high-risk pregnancies. A total of 22/25 foals (88%) presented perinatal asphyxia syndrome; 1/25 (4%) foal had severe flexural deformity, 1/25 (4%) had meconium impaction and 1/25 (4%) had sepsis.

Regarding high-risk pregnancies in the Eutocia Group the median APGAR score was 9 (9–10) and the median gestational length was 340 days (335–345 days). Regarding foal health, there were 12/17 healthy foals (71%) while 5/17 foals (29%) had neonatal encephalopathy; 2/5 foals (40%) needed level 2 care and 3/5 (60%) needed an intensive care regimen (level 3). All mares were discharged, and two foals were euthanized (a foal was euthanized at 40 days of life). In the Dystocia Group, the median APGAR score was 6 (5–8) and the median gestational length was 335 days (330–339 days). Regarding foal health, there were 3/15 healthy foals (20%), 3/15 stillborn (20%), and 9/15 foals (60%) had perinatal asphyxia syndrome complicated by sepsis or other conditions: 2/9 foals (22%) needed level 1 care, 1/9 foal (11%) needed level 2 care and 6/9 (67%) needed an intensive care regimen (level 3).

The mortality rate of the 214 live-born foals was 3.3% (7/214). In particular, the mortality rate in the Eutocia Group was 1.8% (2/165 foals) and in the Dystocia Group was 8.1% (4/49 live-born foals). The 2/7 foals (43%) belonging to the Eutocia Group were subjected to humane euthanasia, due to palatoschisis in one case and due to severe meconium impaction in one case. Of the 4/7 foals (57%) in the Dystocia Group, 2/4 foals died spontaneously, and 2/4 foals were subjected to humane euthanasia due to severe perinatal asphyxia syndrome complicated by other conditions.

### 3.2. Foal Clinical and Blood Parameters in the Control and Dystocia Groups

The healthy foals included in the Control Group were 134/165 foals. Data regarding comparisons between the Control Group and Dystocia Group concerning foal clinical and blood parameters are reported in Table 2, Table 3 and Table 4 respectively. Reported venous blood gas analysis was found in 22 foals of the Eutocia Group, of which only 14 foals met the inclusion criteria for the Control Group.

Considering all quantitative variables, positive but weak correlations were found between duration of stage II and foal weight (R = 0.2; *p* = 0.04), body temperature at birth (R = 0.2; *p* = 0.0001), total number of umbilical coils (R = 0.2; *p* = 0.0035), umbilical coiling index (R = 0.2; *p* = 0.02), foal jugular vein lactatemia (R = 0.3; *p* = 0.0002), pH (R = 0.2; *p* = 0.0001), pCO_2_ (R = 0.2; *p* = 0.0195), and creatine kinase (R = 0.3; *p* = 0.0004). Negative and weak correlations were found between duration of stage II and APGAR score (R = −0.3; *p* = 0.0084), umbilical cord length (R = −0.3; *p* = 0.00145), foal Hct (R = −0.2; *p* = 0.0134), RBCs (R = −0.3; *p* = 0.0275), WBCs (R = −0.2; *p* = 0.0083), neutrophils (R = −0.2; *p* = 0.0079) and HCO_3_^−^ (R = −0.2; *p* = 0.0423).

### 3.3. Descriptive Analysis of the Dystocia Group

A total of 18/57 mares (32%) were primiparous and 29/57 (68%) were multiparous. The incidence of dystocia was not significantly different between primiparous and multiparous mares.

The obstetric interventions for the resolution of dystocia were AVD (45/57; 79%), CVD (5/57; 9%), fetotomy (1/57; 2%), and cesarean section (6/57; 10%). Fetotomy was performed in only one case, in 2004. Cesarean sections were performed at a median gestational age of 340 days (334–345 days), among which there were four stillbirths and two foals born alive, only one of which was discharged from the hospital in good condition after a long period of hospitalization. The mare of this last foal came from a nearby breeding farm and arrived at the facility 30 min after the beginning of the delivery. The foal presented severe flexural deformity of the forelimbs and only the head of the fetus emerged from the vulvar lips. As soon as the mare arrived the EXIT procedure was carried out [24]. The other five mares arrived not earlier than 3 h after the rupture of the chorioallantois. Mares referred for dystocia totaled 5/57 (9%) and the resolutions of dystocia were 1/5 fetotomy, 1/5 CVD and the remaining 3/5 cesarean section. The median time elapsed from the beginning of foaling to dystocia resolution was 300 min (IQR: 190–360 min), with a minimum of 30 min and a maximum of 360 min. The only foal born alive that survived was born after cesarean section, mentioned above, coming from a breeding farm very close by. Furthermore, this mare did not present any postpartum complications.

The categories of dystocia severity in relation to the causes of dystocia, the condition of the foal and mare, and the mortality rate are shown in Table 5. A total of 35/57 dystocias (61%) were categorized as mild dystocia, 10/57 dystocias (18%) were categorized as moderate dystocia and 12/57 (21%) as severe dystocia. Causes of dystocia are shown in Table 6. All parameters (clinical parameters, rapid determinations at foaling and foal venous blood gas, and CBC and blood chemistry parameters) were evaluated in relation to the different causes of dystocia and to the categories of severity. All significant differences among the parameters are illustrated in Table 7 and Table 8.

Since most of the data regarding severe dystocia were lacking, as 6/12 foals were stillborn and some parameters were not recorded in all foals, statistical analysis could not be performed for all variables. Jugular lactate of the foals was higher in severe dystocia (11.8 mmol/L; IQR: 1.0–20.1 mmol/L; N = 3) than in mild (3.8 mmol/L; IQR: 2.7–6.2 mmol/L; N = 30) and moderate dystocia (3.8 mmol/L; IQR: 2.9–5.8 mmol/L; N = 8).

## 4. Discussion

In equine species, fetal survival during dystocia is very short due to the relatively early separation of the placenta; therefore, there can be a high risk of stillbirth [36]. For these reasons, the present study sought to describe not only dystocic delivery in a population of Standardbred mares, but also to evaluate the effects of dystocia on the clinical and blood parameters of their newborn foals.

The incidence of dystocia found in the whole population was 24%. This high percentage is related to the fact that this study was conducted in an equine hospital facility to which mares with high-risk pregnancy, fetal posterior presentation, and/or dystocic delivery initiated on farm are referred by practitioners. Considering only mares with normal pregnancies, the incidence was 4.9%, which is similar to a previous report by Vandeplassche [5] in Standardbred mares. Among mares experiencing dystocia, high-risk pregnancies and gross fetal membrane alterations were more frequent, which could lead to complications during the II stage. In high-risk pregnancies, a suffering or non-viable fetus is more likely to be less active, resulting in inadequate preparation for parturition and/or insufficient active participation in the II stage, subsequently resulting in dystocia due to faulty fetal disposition [12,37,38].

Based on the present results, mares’ age and parity did not influence the frequency of dystocia, in contrast to previous studies that found dystocia to be more frequent in primiparous than in pluriparous mares, especially in older primiparous mares [2,5,39]. It is worth noting that the median age of the mares included in the present case report was nine years; most of them were at their third pregnancy, and older primiparous mares were not present in the population. This is probably because Standardbreds start racing activity at approximately eighteen months of age and debut in the race at two years. Most of them are retired by the age of four and used for reproduction [26].

In the groups studied, the median duration of stage II was longer during dystocia but, due to obstetric intervention, was within the range considered normal for eutocic delivery. Among the causes of dystocia, those characterized by a significantly shorter stage II were those caused by fetal membrane abnormalities, such as in the case of “red bag” delivery, where the intervention consists of the rupture of the chorioallantois [40,41,42]. This condition is easy to diagnose because the velvety-red surface of the intact chorioallantoic sac bulges from the vulva instead of the translucent amniotic sac [40,41,42]. As also stated by other theriogenologists, the process of the chorioallantois detachment can be acute at parturition but may be initiated hours, days, or even weeks prior to delivery, as the disease develops slowly over time [40,41,42]. Moreover, the “red bag” usually results from bacterial infection of the chorioallantois [43,44] and its subsequent thickening or the absence of the thinning process that normally occurs during the last few days of pregnancy [45]. In the present study, red bag delivery was the only representative of a fetal membrane cause of dystocia and, despite the short duration of stage II, all foals born from this type of dystocia showed symptoms referable to hypoxic damage. It is evident that the degree of compromise of foal health when born from a “red bag” delivery depends on the time of chorioallantois separation.

Similarly to previous studies [2,5], the most frequent cause of dystocia among the studied population was of fetal origin, and among these the majority was due to abnormal posture. This result is in agreement with the literature, in which malposture of the extremities accounted for 41% of equine dystocia [6]. Differently from what is reported in Thoroughbred foaling [46], feto-maternal disproportion due to fetal oversize was not observed. In fact, the median foal weight was within normal range for foals of average size. It is not clear if this finding reflects the limited number of observations in the study or an actual breed difference. The low percentage of maternal causes of dystocia was mainly due to primary uterine inertia and incomplete dilatation of the birth canal, in agreement with the literature [2,3,4,5,6,7]. Dystocias resulting from more than one cause were more frequent in mares with high-risk pregnancies (i.e., rupture of the prepubic tendon, severe ventral abdominal hernias, maternal debilitation or prepartum surgery), highlighting the requirement for assisted parturition in facilities with obstetric specialized staff, as also previously suggested [35].

As also observed in cattle [47], there is no common way to describe the level of parturition difficulty in mares. In cattle, some authors suggested different scoring systems to define the level of calving difficulty [47,48,49,50,51,52]. A similar approach was used in the present study, retrospectively dividing all dystocias into mild, moderate and severe foaling difficulty. Severe dystocia was predominantly due to fetal malposition, resulting in higher mortality for both mares and foals than mild and moderate dystocia. Since dystocia raises economic issues and can be a great economic loss for the breeding farm, this categorization could help to provide not only a guide for surviving prognosis but also for hospitalization cost, which would be higher for a mare requiring a cesarean section with her critically ill foal admitted to a neonatal intensive care unit. It would be useful to prospectively create a scoring system to help veterinarians evaluate prognosis for survival and fertility and hospitalization costs.

It is well known that common consequences of dystocia can contribute to a decrease in affected mares’ fertility, but literature is scarce compared to information on cattle. Some studies reported that prolonged attempts at vaginal delivery are associated with multiple complications, including retained fetal membranes (RFM), septic metritis, vaginal trauma, high mortality rates among mares, and decreased foaling rates in subsequent years [53,54]. In the present study, the most frequent consequences were RFM, genital tract lacerations, vaginal hematoma and septic metritis, as similarly reported in previous studies [5,54,55,56,57,58]. A limitation of the present study is that the future fertility of the mares was not evaluated. The information in the Standardbred Stud Book is not reliable for this purpose, since it does not include mares that were pregnant and produced a foal that was not registered in the Stud Book.

Regarding the foals’ health after dystocia, the most common condition presented in this study was Perinatal Asphyxia Syndrome, as previously reported [59,60]. It is well known that the APGAR score is a rapid and valid method to promptly recognize neonates in need of assistance [61,62] and the lower the score, the more severe the health condition of the foal [59]. Results of this study also show that lower APGAR scores were associated with fetal distress and acute hypoxia at birth [59]. Furthermore, foals with lower APGAR were those born from dystocias from more than one cause and from dystocia classified as severe with respect to the type of resolution. The time from birth to first colostrum intake was shorter in foals born after dystocia, despite the delayed appearance of the sucking reflex, because it was administered by medical staff via naso-gastric tube. It is interesting to note that serum IgG levels of these foals were lower than those of healthy foals despite the administration of good quality colostrum. Since hypoxic–ischemic insult can affect the intestinal tract [63], this could cause reduced efficiency of IgG intestinal absorption, as recently suggested [64]. Furthermore, there was a higher frequency of FPT in Group 2, despite medical intervention.

This is the first study that retrospectively evaluated the influence of dystocia on the blood and clinical parameters of the neonatal foal at birth in a dolichomorph breed.

Foal jugular blood lactate was higher after dystocic delivery but within the normal range relative to the whole population [32]. However, in foals born after severe dystocia, blood lactate reached very high values outside the normal range, as reported previously by Kimura et al. [14] in draft horses, showing that metabolic acidosis was directly related to the severity of the dystocia and consequently to the severity of the hypoxic insult. As a consequence, the duration of stage II was positively correlated with blood pH and negatively correlated with bicarbonate, indicating a compensatory response to metabolic acidosis. Unexpectedly, venous blood gas analysis did not show differences between foals born from dystocia and healthy foals born from eutocic delivery, in contrast to previous studies conducted after dystocia in heavy draft foals [14] and in Standardbred foals following induced delivery [65]. This is probably due to the small number of animals (n = 9) for which venous blood gas analysis data were available. It is interesting to note that the two blood gas analyses belonging to foals born from severe dystocia had the lowest pH and bicarbonate values and the highest pCO_2_ values.

In agreement with Chiba et al. [15], RBC counts, Hb levels and Hct were significantly lower in foals born after dystocia, but within the normal range relative to the whole population [66]. Chiba et al. [15] reported normal mean cell volume (MCV) and mean cell Hb concentration (MCHC), indicating possible relative anemia, presumably due to umbilical hemorrhage. It is worth noting that in normal foaling, the mare can remain lying down on her side for up to 30 min after the end of stage II, often with the foal’s hind limbs still inside the birth canal [3,4,66,67,68]. During this period, if the umbilical cord has not yet ruptured, the foal receives blood from the placenta, which is why it may have a higher hematocrit at birth [66,67,68]. In the present study, it can be assumed that the possible cause of the relative anemia in the foals born after dystocia was the low blood volume received from the placenta at the end of stage II parturition. However, it is interesting to note that the lowest values of RBC counts, Hb levels and Hct, outside the normal range, were of foals born from severe dystocia.

The albumin reduction in foals born after dystocia could also be due to possible blood loss at birth or, since albumin is an acute negative phase protein [69], to the inflammatory insult caused by oxygen deprivation. This could also explain the lower number of leukocytes and neutrophils in foals born from dystocia. In fact, among foals born after dystocia, there was only one foal with sepsis, and therefore it seems unlikely that the inflammatory state in the newborns was due to infection. Instead, it seems more likely that the hypoxic insult caused a systemic inflammatory response [70]. The higher serum calcium levels found in foals after dystocia could be due to a decrease in albumin, its main transporter, or due to the onset of Perinatal Asphyxia Syndrome, as previously reported [71].

In the present study, higher CK concentrations were found in foals born after dystocia, but were within the normal range for neonatal foals [72]. This could be due to oxidative stress-induced muscle damage from cholesterol release [15,73,74]. Furthermore, in the present study, CK concentrations were significantly lower in foals born from dystocia due to fetal rather than other causes. The authors can only speculate that faulty fetal presentation/position/posture might not allow wide fetal movement, preventing proper alignment and entry into the birth canal, and therefore fetal muscle resentment could be reduced. In the present study, another parameter found to be the highest in foals born from maternal dystocia was urea, but this was within the normal range for neonatal foals [72]. This finding could indicate, as previously suggested [15], increased protein metabolism due to increased cortisol from fetal stress or could indicate the presence of placental insufficiency [75].

However, hypothetical and speculative conclusions concerning blood parameters need to be critically evaluated in future investigations, since none of these parameters were highly correlated with the type of foaling under the conditions of this study.

In a previous study, probably due to the small number of cases included, the occurrence of dystocia was not statistically associated with amniotic fluid (AF) lactate concentration [20]. Conversely, in the present study, the AF lactate concentration was significantly lower during dystocia. It is well known that AF lactate results from a metabolic and exchange process occurring in the placenta during pregnancy [76]. While in women there is evidence of an increase in AF lactate concentrations in serial measurements during dystocia, caused by intense myometrial activity [77,78,79], an inverse trend seems to exist in mares. Since during pregnancy fetal lactate is supplied by placental production and transfer [80], in agreement with Pirrone et al. [20], this observation could be influenced by the fact that high-risk pregnancies were included in the dystocia group, where placental impairment could have caused a reduced ability to meet fetal metabolic needs through lactate.

Unfortunately, the limited number of non-surviving foals did not allow for statistical analysis highlighting parameters associated with survival or mortality. In the authors’ experience, it seems evident that the time elapsed from the onset of foaling to the obstetric intervention is decisive. For example, in the case of the mare that arrived from a breeding farm located close to the hospital and had her dystocia resolved by cesarean section, the mare did not present any complications and the foal was the only one from referral dystocia that was born alive and survived. Certainly, the opportunity to perform the EXIT procedure also played a crucial role [24] in foal survival.

## 5. Conclusions

In conclusion, the incidence of dystocia in Standardbred mares has a similar incidence compared to other dolichomorphic breeds. However, dystocia cannot be considered only a prolonged stage II parturition, since even a rapid but physiologically abnormal delivery can pose a risk to the life of the newborn and cause complications in the mare. Considering the level of foaling difficulty can help guide the farm and the practitioner to promptly refer mares. Furthermore, since dystocia is more frequent in cases of high-risk pregnancy, it would be recommended to hospitalize high-risk mares at specialized facilities to ensure assisted parturition, possible surgery and intensive care of both mare and foal.

## Figures and Tables

**Table 1:** Mare, fetal membrane and foal information in the Eutocia and Dystocia Groups. For quantitative variables (mare age, parity, gestation length, duration of stages II and III, umbilical cord length, total umbilical coils, umbilical coiling index, and foal weight), data are expressed as median (IQR). For categorical variables (high-risk pregnancy, fetal membrane and umbilical cord alterations, postpartum complications, failure of passive transfer of immunity (FPT), stillbirth, and sick foal), data are expressed as N (%). The different numbers of included animals for each parameter are due to missing data in the clinical reports.

	Eutocia Group (N = 165)	N	Dystocia Group (N = 57)	N	*p*
Mare age (y)	9 (7–12)	165	9 (7–12)	57	
Mare parity (n)	3 (1–5)	165	3 (1–5)	57	
High-risk pregnancy (n)	17 (10%)	165	15 (26% ) #	57	0.002
Gestation length (days)	340 (335–345)	158	340 (334–348)	54	
Duration, stage II (min)	12 (9–15)	155	20 (13–27) *	50	<0.0001
Duration, stage III (min)	45 (30–90)	150	60 (30–120)	42	
Fetal membrane alterations (n)	28 (18%)	155	21 (44%) #	48	0.0003
Umbilical cord alterations (n)	12 (9%)	139	10 (23%)	44	
Umbilical cord length (cm)	54 (47–60)	115	57 (48–69)	36	
Total umbilical coils (n)	5 (4–6)	102	5 (4–6)	33	
Umbilical coiling index	0.09 (0.08–0.11)	102	0.09 (0.08–0.10)	33	
Postpartum complications (n)	30 (18%)	165	25 (44%) #	57	0.002
Foal sex	74 M; 91 F	165	29 M; 20 F	49	
Foal weight (Kg)	47 (43–51)	165	48 (42–54)	57	
Stillborn foals (n)	0	165	8 (14%)	57	
Sick foals (n)	20 (12%)	165	25 (51%) #	49	<0.00001
Level 1 of care	4/20 (20%)		3/25 (12%)		
Level 2 of care	11/20 (55%)		15/25 (60%)		
Level 3 of care	5/20 (25%)		7/25 (28%)		
FPT (n)	7 (5%)	145	8 (23%) #	35	0.0023

* indicates a significant difference between the two groups in the row (quantitative variables). # indicates a significant difference between the two groups in the row (categorical variables).

**Table 2:** Foal clinical parameters and rapid determination performed at foaling in the Control Group (healthy foals) and the Dystocia Group (healthy and sick foals born alive). For quantitative variables (APGAR score, body temperature, time to acquire sternal recumbency, sucking reflex, standing position, first intake of colostrum and rapid determination performed at foaling), data are expressed as medians (IQR). For categorical variables (Failure of passive transfer of immunity (FPT), stillbirths, sick foals), data are expressed as number of subjects (%). Different numbers of included animals for each parameter are due to missing data in the clinical reports.

	Control Group (N = 134)		Dystocia Group (N = 49)		
APGAR score	10 (9–10)	134	8 (6–9) *	49	<0.0001
Body temperature (°C)	37.7 (37.5–37.9)	134	37.6 (37.3–37.9)	46	
Time to sternal recumbency (min)	2 (1–5)	134	4 (1–8)	39	
Time to suckling reflex (min)	20 (12–31)	134	33 (15–56) *	38	0.0044
Time to standing position (min)	68 (57–90)	129	70 (60–92)	36	
Time to first intake of colostrum (min)	116 (95–136)	134	95 (64–130) *^,a^	42	0.0087
Amniotic fluid lactate (mmol/L)	16.3 (14.2–18.4)	102	14.9 (12.8–17.3) *	40	0.0357
Mare jugular lactate (mmol/L)	1.9 (1.3–2.5)	64	2.1 (1.6–2.9)	21	
Foal jugular lactate (mmol/L)	3.1 (2.6–4.2)	128	3.9 (2.8–6.5) *	41	0.0037
Foal jugular glucose (mmol/L)	4.61 (3.77–5.66)	130	4.83 (3.27–5.88)	46	

* indicates a significant difference between the two groups in the row (quantitative variables). ^a^ Foals in the Dystocia Group received colostrum by independent suckling or assisted feeding through a nasogastric tube.

**Table 3:** Foal venous blood gas analysis at birth in the Control Group and the Dystocia Group. Data are expressed as medians (IQR). No significant differences were found. In the Dystocia Group, foals for which venous blood gas analysis was available at birth were born after mild (5/9), moderate (2/9) and severe dystocia (2/9); 3/9 foals (33%) in the Dystocia Group were healthy and 6/9 foals (67%) were sick.

	Control Group (N = 14)	Dystocia Group (N = 9)
pH	7.33 (7.30–7.37)	7.31 (7.26–7.37)
pO_2_ (mmHg)	39 (33–42)	35 (32–40)
SO_2_ (%)	72 (63–75)	65 (60–78)
pCO_2_ (mmHg)	58 (56–60)	59 (53–61)
HCO_3_ (mmol/L)	28.8 (28.3–30.2)	27.2 (25.5–28.1)
Anion gap (mmol/L)	18.4 (17.5–19.5)	17.8 (16.3–19.3)
tCO_2_ (mmol/L)	30.5 (30.2–32.1)	29.3 (27.4–31.3)
Base excess (mmol/L)	2.0 (0.86–3.0)	−0.70 (−2.77–1.77)
Na^+^ (mmol/L)	151 (146–152)	150 (146–152)
K^+^ (mmol/L)	3.7 (3.4–4.2)	4.1 (3.6–4.4)
Cl^−^ (mmol/L)	107 (105–107)	106 (106–109)

**Table 4:** Foal CBC and blood chemistry at birth in the Control Group and the Dystocia Group. Data are expressed as medians (IQR) and min–max values. Different numbers of included animals for each parameter are due to missing data in the clinical reports.

	Control Group	N	Dystocia Group	N	*p*
Hb (g/dL)	15.5 (14.8–16.1) 13–17.8	134	14.6 (13.7–15.8) * 8.6–16.8	47	0.005
Hct (%)	46.2 (44–49) 39–56	134	44.8 (41.2–47.9) * 24.2–52.1	47	0.0247
RBCs × 10^3^/mm^3^	10,920 (10,423–11,500) 8050–99,500	134	10,370 (9790–10,868) * 6380–12,370	47	0.0002
Platelets × 10^3^/mm^3^	207 (174–241) 64–724	134	194 (163–232) 89–305	47	
WBCs/mm^3^	7910 (6972–9032) 4610–13,310	134	7290 (6175–8167) * 3600–12,340	47	0.0050
Neutrophils/mm^3^	6205 (5135–7013) 2800–11530	126	5430 (4622–6291) * 1080–10740	47	0.0029
Lymphocytes/mm^3^	1350 (1150–1601) 590–3256	126	1390 (1098–1915) 330–3390	47	
CK (U/L)	187 (142–243) 52–525	114	262 (183–377) * 84–1218	40	0.0007
Total Bilirubin (µmol/L)	35.91 (29.07–44.46) 0.7–4.7	114	35.91 (29.07–41.04) 1.1–3.8	40	
Triglycerides (mmol/L)	0.11 (0.08–0.12) 2–24	26	0.1 (0.09–0.15) 5–21	46	
Total Protein (g/L)	42 (40–44) 1.8–7.8	116	40 (38–43) 1.4–5.2	18	
Albumin (g/L)	33 (32–35) 2.7–4.3	116	32 (29–34) * 2.5–3.8	46	0.0126
Albumin/Globulin	4.1 (3.6–4.7) 0.8–7.96	116	3.7 (3.3–4.3) 1.22–6.63	46	
Urea (mmol/L)	13 (11.39–14.64) 19.9–40.7	118	12.78 (10.78–14.21) 24–54.5	46	
Creatinine (µmol/L)	221.05 (194.52–256.42) 1.3–4.0	118	238.73 (194.52–318.31) 1.6–14.4	46	
Phosphorus (mmol/L)	1.74 (1.58–1.87) 3.9–6.9	27	1.8 (1.55–1.93) 4.0–6.3	18	
Calcium (mmol/L)	3.22 (3.15–3.32) 10.9–17.0	114	3.35 (3.2–3.57) * 11.2–17.2	40	0.0032
Magnesium (mmol/L)	0.73 (0.69–0.78) 1.1–2.8	114	0.73 (0.69–0.82) 1.5–3.5	45	
Fibrinogen (g/L)	3.2 (1.7–4.1) 0.43–5.1	108	2.0 (1.7–3.2) 1.0–4.9	43	
IgG within 24 h (mg/dL)	1659 (1144–2315) 752–3500	116	900 (827–1440) * 307–2315	38	0.0001

* Indicates a significant difference between the two groups in the row.

**Table 5:** The three categories of dystocia severity in relation to cause of dystocia, condition of the foal and mare, and mortality rate.

Category of Dystocia Severity % N	Cause of Dystocia	Foal Condition	Postpartum Complications in Mares	Mortality Rate
Mild 61.4% (N = 35)	Maternal (9/35)	Healthy 20/35 Sick 15/35 Stillborn 0/35	15/35	Mares 6% (2/35) Foals 6% (2/35)
	Fetal (17/35)			
	Fetal membranes (6/35)			
	More than one (3/35)			
Moderate 17.5% (N = 10)	Maternal (1/10)	Healthy 3/10 Sick 5/10 Stillborn 2/10	3/10	Mares 0% Foals 20% (2/10)
	Fetal (3/10)			
	Fetal membranes (1/10)			
	More than one (5/10)			
Severe 21.1% (N = 12)	Maternal (1/12)	Healthy 1/12 Sick 5/12 Stillborn 6/12	3/12	Mares 25% (3/12) Foals 67% (8/12)
	Fetal (9/12)			
	Fetal membranes (0/12)			
	More than one (2/12)			

**Table 6:** Causes of dystocia and information regarding stillbirth and foal and mare mortality. Data are expressed as % (n).

Causes of Dystocia % (n)	Specific Causes % (n)	Stillbirth % (n)	Foal Mortality % (n)	Mare Mortality % (n)
Maternal causes 19.3% (11/57)	Primary uterine inertia 45.5% (5/11) [13,23]	0%	0%	0%
	Weak abdominal straining 36.4% (4/11) [35]			
	Incomplete cervical dilation 18.2% (2/11) [35]			
Fetal causes 51% (29/57)	Abnormal fetal posture 72.4% (21/29)	21% (6/29)	6.9% (2/29)	6.9% (2/29)
	Abnormal fetal position 17.2% (5/29)			
	Abnormal fetal presentation 7% (2/29)			
	Congenital deformities 3.4% (1/29)			
Fetal membrane causes 12.3% (7/57)	Premature placental separation 86% (6/7)	0%	14.3% (1/7)	14.3% (1/7)
	Umbilical cord twisting 14% (1/7)			
More than one cause 17.5% (10/57)	Abnormal fetal position and secondary uterine inertia 30% (3/10)	20% (2/10)	10% (1/10)	20% (2/10)
	Premature placental separation and abnormal fetal presentation 20% (2/10)			
	Incomplete cervical dilation and abnormal fetal position 10% (1/10)			
	Weak abdominal straining and abnormal fetal posture 10% (1/10)			
	Premature placental separation, secondary uterine inertia, and abnormal fetal position 10% (1/10)			
	Abnormal fetal posture and secondary uterine inertia 10% (1/10)			
	Premature placental separation and secondary uterine inertia 10% (1/10)			

**Table 7:** Significantly different parameters among the different causes of dystocia reported. Data are expressed as medians (IQR) and min–max values.

	Maternal Causes (n = 11)	Fetal Causes (n = 29)	Fetal Membrane Causes (n = 7)	More Than One Cause (n = 10)	*p*
Duration of stage II (min)	21 (18–25) ^a^ 8–75 N = 11	22 (14–34) ^a^ 5–360 N = 24	7 (4–11) ^b^ 3–14 N = 4	26 (14–50) ^a^ 10–65 N = 10	0.0386
APGAR score	9 (8–10) ^a^ 8–10 N = 11	8 (7–9) ^b^ 3–10 N = 27	8 (5–9) ^bc^ 5–9 N = 7	6 (2–7) ^c^ 0–9 N = 9	0.0011
Foal CK (U/L)	339 (223–449) ^a^ 109–827 N = 11	207 (144–271) ^b^ 84–395 N = 18	397 (320–447) ^a^ 303–486 N = 5	323 (237–454) ^a^ 158–1218 N = 6	0.0185
Foal urea (mg/dL)	40 (36–48) ^a^ 34–55 N = 11	34 (29–38) ^b^ 24–48 N = 21	34 (30–39) ^b^ 29–48 N = 7	31 (29–37) ^b^ 25–44 N = 7	0.0193

^a, b, c^ Different superscript letters indicate a significant difference among the four groups in the row.

**Table 8:** Significantly different parameters among the different categories of dystocia severity reported. Data are expressed as medians (IQR) and min–max values. A total of 35/57 foals were born from Mild dystocia (20/35 foals were healthy; 15/35 foals were sick); 10/57 foals were born from Moderate dystocia (2/10 foals were stillborn, 3/10 foals were healthy and 5/10 foals were sick); 12/57 foals were born from Severe dystocia (6/12 foals were stillborn, 1/12 foals were healthy and 5/12 foals were sick).

	Mild Dystocia (n = 35)	Moderate Dystocia (n = 10)	Severe Dystocia (n = 12)	*p*
Duration of stage II (min)	16 (11–24) ^a^ 3–41 N= 35	25 (22–30) ^b^ 12–50 N= 9	75 (62–112) ^c^ 55–165 N= 5	0.0001
APGAR score	9 (8–9) ^a^ 6–10 N= 35	5 (3–8) ^b^ 0–9 N= 10	3 (0–5) ^b^ 0–8 N= 6	<0.0001
Foal Hb (g/dL)	14.5 (13.7–15.4) ^a^ 8.6–16.8 N= 35	16 (14.9–16.2) ^a^ 14.5–16.4 N= 8	13.4 (11.3–14.2) ^b^ 11.1–14.5 N= 5	0.0033
Foal Hct (%)	44.9 (41.3–47.4) ^a^ 24.2–52.1 N= 35	44.8 (43.4–51.1) ^a^ 40.8–51.9 N= 8	39.9 (36.3–41.5) ^b^ 33.6–42.5 N= 5	0.0048
Foal RBCs × 10^3^/mm^3^	10375 (9898–10861) ^a^ 6380–12370 N= 35	11095 (10286–11807) ^a^ 8500–11980 N= 8	8700 (8283–9390) ^b^ 8130–9790 N= 5	0.0044
Foal total proteins (g/dL)	4.1 (3.9–4.3) ^a^ 1.4–5.2 N= 35	4.2 (3.7–4.3) ^ab^ 3.4–4.4 N= 8	3.5 (3.2–3.9) ^b^ 3.2–3.9 N= 4	0.0417
Foal calcium (mg/dL)	13.1 (12.6–13.6) ^a^ 11.2–17.2 N= 30	13.8 (13.3–14.3) ^ab^ 12.9–14.6 N= 7	14.7 (14.4–15.1) ^b^ 14.3–15.2 N= 3	0.0141

^a, b, c^ Different superscript letters indicate a significant difference among the three groups in the row.

## Data Availability

The data presented in this study are available on request from the corresponding author.
